# Tare Soil Disinfestation from Cyst Nematodes Using Inundation

**DOI:** 10.3390/life13010057

**Published:** 2022-12-24

**Authors:** Beatrice Berger, Matthias Daub, Kathleen Gärtner, Matthias Becker, Stephan König

**Affiliations:** 1 Julius Kühn Institute (JKI)—Federal Research Centre for Cultivated Plants, Institute for Plant Protection in Field Crops and Grassland, 38104 Braunschweig, Germany; 2 Julius Kühn Institute (JKI)—Federal Research Centre for Cultivated Plants, Institute for Plant Protection in Field Crops and Grassland, 50189 Elsdorf, Germany; 3 Great Ormond Street Institute of Child Health, University College London (UCL), London WC1N 1EH, UK; 4 Julius Kühn Institute (JKI)—Federal Research Centre for Cultivated Plants, Institute for National and International Plant Health, 38104 Braunschweig, Germany

**Keywords:** tare soil residues, potato and sugar beet cyst nematodes, Brukner basin, sedimentation basin, lactic acid, phytosanitary measures

## Abstract

The dissemination of soil tares in the potato and sugar beet processing industry is one of the main paths for the spread of potato cyst nematodes (PCN), a severe quarantine pest. Efficient measures for the disinfestation of tare soil from PCN, but also from beet cyst nematodes (BCN), are needed. In our study, *Globodera pallida* (a PCN) and *Heterodera schachtii* (a BCN) cysts were sealed in gauze bags and imbedded in sedimentation basins. The cysts were either placed (a) in a presedimentation basin (Brukner basin) for three days, (b) in the presedimentation basin for three days and subsequently in sedimentation basins for nine weeks or (c) in sedimentation basins for nine weeks (without presedimentation). We tested the viability of the eggs and juveniles by hatching assays and using the reproduction rates in bioassays. We demonstrated that PCN and BCN imbedded in a sedimentation basin were only still showing some hatching activity after 2.5 weeks, while no hatching was observed when an additional Brukner basin treatment was conducted before sedimentation.

## 1. Introduction

Potato cyst nematodes (PCN) (*Globodera pallida* and *G. rostochiensis*) and beet cyst nematodes (BCN) (*Heterodera schachtii*) are severe threats to agriculture. Even though the host range for PCNs is narrow, comprising only species of the genus *Solanum*, BCN is associated with several dicotyledonous species mainly from Chenopodiaceae and Cruciferae. Both pests are an economic threat to potato plants (*Solanum tuberosum* L.) and sugar beets (*Beta vulgaris*).

Without managing strategies, losses caused by PCNs in potatoes can range between 10 and 100% depending on the resistance of the potato variety [1] and losses caused by BCN in sugar beets can range between 20 and 50%. To stop the spread of cyst nematodes complex management strategies are needed, including the breeding of resistant cultivars, crop rotation and other preventive measures. In particular, the durable nature of the female cyst filled with viable eggs and juveniles poses a high phytosanitary risk [2]. Cysts with vital eggs can persist several years in the soil and, therefore, soils adhering to potato tubers and sugar beets are a potential source of spread during harvest and further processing.

Panagos and colleagues showed in 2019 that the total loss of soil during crop harvest was 14.7 million tons per year in the EU, where potatoes and sugar beets account for 65% [3]. Soils adhering to potato tubers and sugar beets could be returned to arable land after being processed, to minimise soil losses. However, before reusing the valuable soils, cyst nematodes need to be eliminated.

There are different approaches to tackle this problem. One is the process of inundation, where the soils are completely covered with water for a certain amount of time to disinfest them [4,5]. Inundation has been shown to control nematodes like root-knot nematodes (*Meloidogyne* spp.) [6] or stem nematodes (*Ditylenchus dipsaci*) [7]. In flooded peat soil, the viability of eggs and juveniles of *G. pallida* cysts were reported to be significantly reduced (97.7–99.7%) [5]. Oxygen stress is known to inhibit the hatching of juveniles. Depending on their spatial position in the cysts, oxygen levels vary and therefore different grades of hatching and delayed development of juveniles in the cysts can be observed [8]. Although oxygen depletion during the flooding is highly lethal for cyst nematodes some of them may survive the inundation process, as it is complex and affected by biotic and abiotic factors. It is known that the percentage of lethality strongly depends on the duration of flooding [9,10] and temperature [11]. Mesophilic-anaerobic conditions, for example, result in the deactivation of vital eggs of *G. rostochiensis* after six to nine weeks (35 °C), whereas *G. pallida* appears to be more resistant under these conditions [11].

Other stressful conditions for nematodes, such as the reduction of the redox potential and soil pH, denitrification (chemical reduction of nitrate to molecular nitrogen N_2_), accumulation of ammonia, chemical reduction of sulphates and production of organic acids (e.g., methane and hydrogen sulphide), were described by Inglett and colleagues [12]. The production of compounds toxic to nematodes could be due to changes in the microbial community in the sedimentation basins, which has been shown to inactivate fungi and bacteria [13,14]. The sugar beet processing industry already uses sedimentation basins to store tare soils after beet processing. The organic material in the processed soil consists of abrasion and fragments from the beet washing. Tare soil from potato processing is dry and usually contains less organic material compared to the soils from sugar beet processing. Therefore, the implementation of flooding sedimentation basins in the potato processing industry might be more complex. In addition, the minimum period of inundation for the disinfestation of tare soils is very important, because cultivation circles in sugar beet and potato-growing industries are tight, and plant health and economic interests need to be considered. Unlike composting processes or the heat treatment of residual soil (100 °C or 70 °C), the inundation of tare soil is not yet considered a recommended treatment within regulatory aspects of phytosanitary regulation for the control of PCNs [15], although the nongovernmental European and Mediterranean Plant Protection Organization (EPPO) included the inundation procedure in a revised guidance very recently [16].

In some sugar beet processing factories, a presedimentation basin called the Brukner basin is installed as an upstream facility to the sedimentation basins. How the Brukner basin contributes to the success of disinfestation is not known yet. Primarily, the Brukner basin recirculates the purged wash water back to the cleaning process of beets and, at the same time, helps to concentrate and reduce the volume of wash water containing tare soil residues. By adding slaked lime to the wash water, the pH in the Brukner basin rises to about 11. Only during the regular monthly cleaning processes to remove precipitated calcium carbonate from the pipe system does a rapid pH decrease to pH 5.5 occur for two days in the Brukner basin. It would be important to determine whether additional treatment in a Brukner basin could accelerate the disinfestation process or whether the basin itself might be sufficient to devitalise PCN and BCN to optimise inundation processes in the potato processing industries.

Therefore, the aim of this study was to determine the viability and reproducibility of *G. pallida* and *H. schachtii* cysts after treatment in sedimentation basins and the role of an additional Brukner basin during the process. We also analysed the roles of calcium hydroxide (slaked lime) and lactic acid, the latter as an intermediate product in microbial carbon hydrate metabolism, and the influence of the inundation of different soil types on PCN viability under laboratory conditions. The results of this study are intended to provide guidance to the plant protection authorities for the evaluation of inundation as a treatment process with regard to the reliable inactivation of PCNs, as one possible treatment required by the currently enacted EU commission implementing regulations [17].

## 2. Materials and Methods

### 2.1. Cyst Nematode Propagation

For our inundation studies, we used reference PCN and BCN cyst material of defined nematode populations. We chose *Globodera pallida* (Pa2 “Kalle”) and multiplied the cysts on susceptible potato cultivar “Desiree” in the climate growth chamber facilities of the Julius Kühn Institute (JKI) at 20 °C/18 °C (16 h/8 h, respectively) in 2018 for the sedimentation basin experiments. In 2020, a second set of *G. pallida* reference cysts was produced for the in vitro experiments under greenhouse conditions. To mimic a PCN-typical diapause, prior to further use all newly formed cysts were kept at 4 °C for at least three months. *Heterodera schachtii* (Pa Schach0 “MS”) cysts were propagated on highly susceptible winter oilseed rape cultivars in the greenhouse at JKI field station in Elsdorf, Germany. *Heterodera schachtii* second-stage juveniles (J2s) were acquired after incubation of the cysts for seven days in a 5-mMol ZnCl_2_ solution to induce hatching. Pots with oilseed rape seedlings were then inoculated with these J2s and newly formed cysts were harvested after the development of two generations at approximately 20 °C in 2018, as described in Daub et al., 2020 [18].

### 2.2. Test of Different Soil Types during Inundation

To test the impact of soil type on the success of inundation, we chose two contrasting soil types, sand with high particle size > 0.2 mm and clay loam soil with medium/low particle size between 0.6 µm–2 mm. The soils were then either left dry or saturated with water and incubated with open (aerobic condition) or closed lids (anaerobic condition). Twenty cysts per condition were incubated for two weeks in four replicates. As the control for dry conditions, the PCNs were incubated in plastic vials and, for wet conditions, in water only.

### 2.3. Inundation Experiments

**Figure 1:**
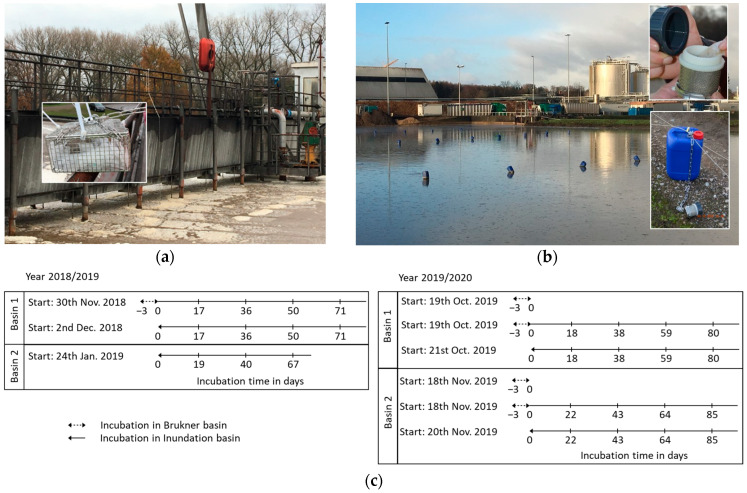
Experimental set-up at the sugar factory in 2018/2019. The Brukner basin (**a**) is up-stream of the sedimentation basins. One subset of *Globodera pallida* and *Heterodera schachtii* cysts samples, fixed in a metal basket (small picture), was placed for three days in the Brukner basin. Subsequently, the subset from the Brukner basin and another new cyst set were installed together in the sedimentation basin (**b**) for 17, 36, 50 and 71 days using little metal baskets with screw lids ((**b**) small pictures) that were attached to plastic swimmers (blue containers) on steel wire ropes. (**c**) Time frame of sampling during period 2018/2019 and 2019/2020.

The inundation experiments (2018–2019 and 2019–2020) were started shortly after the beginning of the sugar beet campaigns in the sedimentation basin system of a sugar factory in Uelzen, Northern Germany. The sedimentation basin system included two preconditioning Brukner basins (Figure 1a) and three sedimentation basins at the time of the experiments (Figure 1b,c). The sedimentation basins had a capacity of 32,990 m^3^, 32,850 m^3^ and 10,350 m^3^ and were connected to each other. This is necessary for pumping variable sludge volumes so as to enable the exchange of sludge and separate the water from the solid particles. The daily amount of processed beets was >10,000 tons (t), which resulted in the production of approximately 80,000 t of tare soils during the five months of the campaign. Due to the low amount of processed sugar beets, only one preconditioning Brukner basin was operated in the 2018/2019 and in the 2019/2020 campaign.

We set up two independent sets of experiments, one in 2018/2019 and one in 2019/2020. The filling of the first basin with sludge water in 2018 started on 21 September, and on 20 December for the second basin. In 2019, the filling of the first sedimentation basin started on 19 September and of the second one on 2 November.

Subsets of *G. pallida* and *H. schachtii* reference cysts were introduced into the Brukner basin (Figure 1a) on 30 of November 2018. In 2019, samples were introduced into the Brukner basin on 19 of October. In addition, cysts were treated only in the sedimentation basin (starting 24 January 2019). Samples were taken at different time points and the time courses are shown in Figure 1c.

To ensure that our reference cysts were not released into the environment, they were sealed in permeable nylon gauze sachets (2 × 2 cm, 100 µm mesh size). Each sachet contained 100 reference cysts and was packed into another gauze sachet with a 200 µm mesh size. In total, 20 sachets with PCN and 20 sachets with BCN cysts were prepared. The sachets were placed in a metal cage (30 cm × 15 cm × 15 cm) which was fixed on a metal chain (Figure 1a) in the Brukner basin 1 m below the surface. After three days, one of each of the cyst samples was transferred to the sedimentation basin. We prepared individual metal baskets for each time point, which also contained PCN and BCN cyst sachets that were not exposed to the Brukner basin (Figure 1b,c). The metal baskets were held one meter below the surface by attaching them to 10 L plastic swimmers. They were held in place in the basin using wires, fixed to pegs, at a distance of 3 × 10 m. Two sensors were installed at a depth of 1 m below the surface to monitor the temperature, pH-value and oxygen content. Five metal baskets were analysed for each time point. The pH-value was stable at pH ~6.5 (Supplementary Figure S1). The oxygen level was close to zero (Supplementary Figure S2) and water temperature depended on the air temperature (Supplementary Figure S3).

After collection of the samples, they were cleaned with water to remove adherent soil residues. PCN cysts were air dried for three days in the laboratory before further analysis. Sachets of BCNs were either transported in 1 g of moist loess-clay or wrapped in moist kitchen roll. The samples were then shipped to the lab overnight in a closed container. PCN and BCN samples were analysed for their hatching activity (see Section 2.4) and their ability to reproduce on susceptible host plants (see Section 2.5).

In another factory of the company with similar facilities, the sugar contents and organic acids were determined at different sites in the inundation waters of a sedimentation basin in 2021. Concentrations were analysed with a HPLC-based method adapted by the company. In short, the different carbon hydrates were separated on a Ca column (Aminex HPX-87C column, BioRad, Feldkirchen, Germany) with a flowrate of 0.7 mL min^−1^ and a temperature of 80 °C. Water served as an eluent. Whereas organic acids were separated on an H^+^ column (Aminex HPX-87H column, BioRad, Feldkirchen, Germany) with a flowrate of 0.6 mL min^−1^ and a temperature of 36 °C. A 1.25 mmol L^−1^ diluted sulphuric acid solution served as the eluent. A refractive index detector detected the substances.

### 2.4. Hatching Test

Hatching tests for treated and control PCN cysts were performed according to EPPO Standard PM 7/40 [19]. The tests demonstrated the ability of juveniles to eclose from eggs within the cysts. Twenty randomly chosen cysts per treatment and replicate were exposed to 1 mL potato root diffusate (v:v: 1:2 root diffusate:water) collected from the susceptible potato cultivar “Desiree” (Agropa GmbH, Brunnen, Germany). Newly emerged juveniles were counted weekly under a binocular (WILD Heerbrugg AG, Heerbrugg, Switzerland) and the diffusates were renewed. After seven weeks, the sum of hatched juveniles per cyst was calculated and compared to the hatch of nontreated control cysts.

From the same samples, the PCN cysts were visually analysed for deformation and documented using a stereomicroscope Axio Zoom V16 connected to an Axiocam (Carl Zeiss Microscopy, Jena, Germany). In addition, *G. pallida* cysts from three weeks (inundation treated and nontreated controls) were cut to release the eggs. The eggs from the cysts were stained with malachite green solution (v:v, 1:5) for 15 min (Sigma-Aldrich, Taufkirchen, Germany) and examined under a microscope (Leica CTR 5500, Leica Microsystems, Wetzlar, Germany) to determine morphological changes of the juveniles in the eggs.

For BCN hatching tests, 20 cysts were randomly selected from the gauze bags and placed on petri dishes filled with 5 mmol ZnCl_2_ solution for 14 days at room temperature. Subsequently, the juveniles were separated using a 20 µm sieve and counted under a stereo microscope (Leica MZ APO, Leica Microsystems, Wetzlar, Germany) at 50× magnification. Unhatched juveniles and inactive eggs were isolated from the remaining cysts using a cyst homogenizer [20] before counting. Hatching ratios were calculated from the number of hatched vs. number of unhatched nematodes.

### 2.5. Reproduction Test (Bioassays)

For PCN, the susceptible potato cultivar “Desiree” was pregerminated in the dark at 20 °C for ten days until the first sprouts were visible. The germinated tubers were transferred to Teku^®^ pots (9 × 9 × 10 cm) (Pöppelmann GmbH & Co. KG, Lohne, Germany) that were half filled with loess-loam that was almost nutrient free and free from soil fauna. The loess-loam was supplemented with 1.5 g kg^−1^ Osmocote^®^ Exact Standard 5-6M (ICL Speciality Fertilizers, Nordhorn, Germany) to ensure optimal plant nutrition. For each pot, one gauze sachet with 50 treated or control reference cysts was placed next to the tuber. Next, the pots were filled up with the loess-loam free substrate and placed on single trivets to avoid nematode transfer between the pots. Over a period of 15 weeks, plants were kept at 20 °C/16 °C for 16 h/8 h (day/night settings, respectively) under natural light conditions, and were watered on demand. During that time, soil temperature was recorded to ensure that a required daily temperature sum of 1800 °C was achieved, which is necessary to complete the life cycle of 1 to 1.5 PCN generations [15]. At this point, the aboveground plant tissue was cut off and the substrate in the pots was removed by washing through a 250 µm sieve (RETSCH GmbH, Haan, Germany), which held back the newly formed cysts. The number of cysts was counted and the formation rate was calculated as P_f_/P_i_ quotient, where P_f_ represents the final number and P_i_ the initial number of cysts present in the pot. A P_f_/P_i_ quotient higher than one represents an increase of cysts, indicating population growth. Five replicates per treatment with 50 cysts per pot were used.

In the case of BCN, the nylon gauze sachets containing 50 treated or nontreated (control) cysts that were placed in a 400 mL pot and filled with 350 g of loess enriched with fertilizer (3 g kg^−1^ Osmocote Exact 3–4 M). Four seeds of a susceptible oilseed radish cultivar (“Siletina”) were sown per pot and thinned to two remaining plants after the seedling emerged. Each pot was treated with 30 mL of the fungicide Previcur (1.5 mL L^−1^, 530 g L^−1^ propamocarb, and 310 g L^−1^ fosetyl), applied as a drench, to prevent seed-borne diseases. Greenhouse condition were set up in a randomised block design at 20 °C/16 °C for 16 h/8 h (day/night settings, respectively), and additional light (2700 K) to sun light was provided for 12 h. The data logger continuously recorded soil temperature and the test was terminated after 390 dd10 (sum of the days with a temperature >10 °C) allowing the reproduction of one *H. schachtii* generation. This value was determined by specific controls with untreated cysts that achieved reproduction rates of P_f_/P_i_ > 2. After removing the original gauze bags used for inoculation, newly developed cysts were extracted from loess from individual pots using a sieve combination (1000/100 µm) and a subsequent centrifugal flotation method as described before [21]. The cysts were counted and the contents (juveniles and eggs) were released from the cysts using a cyst homogenizer. Juveniles and eggs were quantified per 100 g of soil using a stereo microscope at 50× magnification. The reproduction rate per pot was calculated as P_f_/P_i_, where P_i_ was the number of 50 cysts initially placed in each pot and compared to controls with untreated cysts that achieved reproduction rates of P_f_/P_i_ > 2.

### 2.6. PCN Cysts Exposed to Slaked Lime and Different pH-Values

In the manufacturing process of refined white sugar, calcium hydroxide (slaked lime) is used to remove hemicellulose fractions from the sugar juice. This is done to facilitate crystallisation of the resulting clear, thin juice. The slaked lime is therefore added to the washing water before further processing of the beets and extraction of the sugar juice in order to minimise sugar losses through enzymatic processes that lead to the unwanted breakdown of sucrose. Consequently, tare soils with nematode cysts are also exposed to slaked lime. Since we were not able to introduce our reference cysts in the whole sugar beet processing, we simulated the slaked lime treatment under laboratory conditions to evaluate the impact on PCN viability. Five hundred mL plastic flasks with screw lids were filled with either 150 mL sandy soil or clay loam soil. Then 200 mL lime solution was added, and the pH-value was adjusted to 11 by adding 95% sulphuric acid (AppliChem GmbH, Darmstadt, Germany). Four gauze sachets per soil type, each with 15 cysts per sachet, were then placed in the soil layer of the respective flask for four days. After four days, gauze sachets were transferred from strong alkaline conditions to plastic flasks containing soil tare with pH 7 and the cysts were kept there for another two days. In a second treatment, we started with pH 7 for four days and the cysts were then placed into pH 11. After the treatments, ten cysts were exposed to potato root diffusates and the hatching activity was determined. The experiment was repeated twice.

To test the influence of different pH-values on PCN in the laboratory we prepared potassium hydroxide solutions with pH 7.8, 9, 10, 11 and 12 and autoclaved tap water as control. Twenty reference cysts were added to each glass vial, with three replicates per pH-solution. After 24 h, the cysts were removed from the solutions, and hatching assays, with ten cysts per treatment and replicate, were conducted.

### 2.7. PCN Cysts Exposed to L-Lactic Acid

Under anaerobic conditions, as given in the inundation process, bacteria can produce lactic acid. In order to test the impact of lactic acid on the viability of eggs and juveniles in *G. pallida* cysts, we treated the cysts with 2.5%, 5% or 10% of L-lactic acid (Carl Roth GmbH, Karlsruhe, Germany). Twenty intact *G. pallida* cysts were placed in a 10 mL glass vial containing 5 mL of L-lactic acid. Autoclaved tap water served as a control. The treatments were done with five replicates and the experiment was repeated twice. Cysts were exposed to the solution for seven days at room temperature before they were removed from the solution and the hatching activity was determined.

### 2.8. Anaerobic and Aerobic Micro Flooding of PCN in Different Soils

To test the impact of soil type on the success of inundation, we chose two contrasting soil types, clay loam soil with medium (0.2–2.0 mm) to low (0.6–2.0 µm) particle sizes and sand with a high (>0.2 mm) particle size. Five hundred mL plastic vials were filled with either 200 g clay loam soil or sand. An additional eight vials with sand or clay loam soil were watered with 100 mL water. Four of the soil-filled and four of the water-filled vials were screwed tight to simulate additional oxygen depletion, the other four respective vials were left open. As control, we used four open vials filled only with water. To evaluate the impact of inundation, we also prepared four open lid vials with sand or brown soil without adding water on top. Twenty *G. pallida* cysts sealed in gauze sachets were placed in the middle of the vials and rested for two weeks. Subsequently, they were washed and ten cysts per treatment and replicate were analysed in a hatching test once a week over a period of six weeks. Nontreated *G. pallida* cysts served as external control of the hatching activity of eggs and juveniles. The experiment was repeated twice.

### 2.9. Statistics

The data were analysed using the R Version R-4.03 with visualisation in the RStudio packages stats and ggplot2. Data from the hatching tests and bioassays were analysed using non-parametric Kruskal-Wallis tests with pairwise Wilcoxon post hoc tests using adjusted *p*-values, in case data did not fit a normal distribution according to the Shapiro-Wilk test. A Pearson correlation coefficient was calculated for the correlation between L-lactic acid concentration and *G. pallida* hatching activity using the ggpubr package [22]. In the KOH test as described under 2.5, data were normally distributed and a one-way ANOVA was performed.

## 3. Results

### 3.1. Influence of Oxygen, Soil Type and pH-Values on PCN and BCN Cysts In Vitro

#### 3.1.1. Inundation Reduces Viability of *G. pallida* Cysts Independent of Soil Type

Arable land is usually characterised by various soil types with different particle sizes and mineral content. We tested sand and clay loam soil under wet and dry conditions to determine whether they show differences in the efficiency of cyst nematode inactivation.

**Figure 2:**
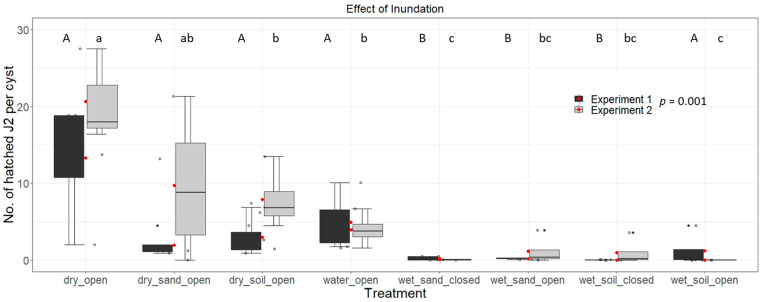
Hatching test of *Globodera pallida* cysts after inundation in water, sand or brown soil with and without exclusion of oxygen. Total number of hatched second stage juveniles (J2s) per cyst exposed to potato root diffusates after a period of six weeks are shown from two independent experiments. Beforehand, 20 cysts per treatment and replicate were placed in 500 mL plastic vials. Cysts were embedded as follows for two weeks: 100 mL of water, no lid (wet_open), 200 g of sandy soil without water, no lid (dry_sand_open), 200 g of clay-loam soil without water, no lid (dry_soil_open), 200 g of sandy soil with 100 mL of water, no lid (wet_sand_open), 200 g of clay-loam soil with 100 mL of water, no lid (wet_soil_open), 200 g of sandy soil with 100 mL of water, with lid (wet_sand_closed), 200 g of clay-loam soil with 100 mL of water, with lid (wet_soil_closed) and nontreated control cysts (dry_open). Data were analysed using non-parametric Kruskal-Wallis tests with pairwise Wilcoxon post hoc tests. Box-Whisker-plots display median and mean (red dots) values of four replicates per treatment. Different letters indicate significant differences between the treatments of the respective experiment.

We show that incubation in dry soil already has the ability to reduce the hatching activity of *G. pallida* cysts compared to cysts in plastic vials (Exp.1: 13.3 hatched J2s per cyst; Exp.2: 23 hatched J2s per cyst). The results for clay loam soil were: Exp.1: 13.3 hatched J2s per cyst; and Exp.2: 23 hatched J2s per cyst; and for sand: Exp.1: 2.9 hatched J2s per cyst; and Exp.2: 7.9 hatched J2s per cyst (Figure 2). It was almost completely abolished in wet clay loam soil (Exp.1: 1.2 hatched J2s per cyst; Exp.2: 0 hatched J2s per cyst) and in wet sand (Exp.1: 0.2 hatched J2s per cyst; Exp.2: 1 hatched J2s per cyst). The presence (open lid) or absence (closed lid) of oxygen did not increase the inactivation of the cysts further (Figure 2).

#### 3.1.2. Hatching Activity of *G. pallida* Is Not Affected by Alkaline pH-Values

The cleaning of the Brukner basin during potato and sugar beet processing leads to a drop in pH, leading to the tare soil being exposed to different pH-values, ranging from 6 to 11. To test whether this has an influence on the hatching activity of *G. pallida*, we prepared KOH-solutions ranging from pH 7.8 to 12, using autoclaved tap water as a control (pH 7.2, see Table 1). Twenty cysts per condition were incubated in triplicates for 24 h and viability was tested using the hatching assay. We showed that basic solutions (pH > 7.2) do not have an influence on the hatching capacity of PCNs compared to the water control. We saw a slight increase in hatching second stage juveniles (J2s) at pH 12, but this was not significantly different from the control treatment.

#### 3.1.3. L-Lactic Acid Inhibits the Hatching Activity of *G. pallida*

**Figure 3:**
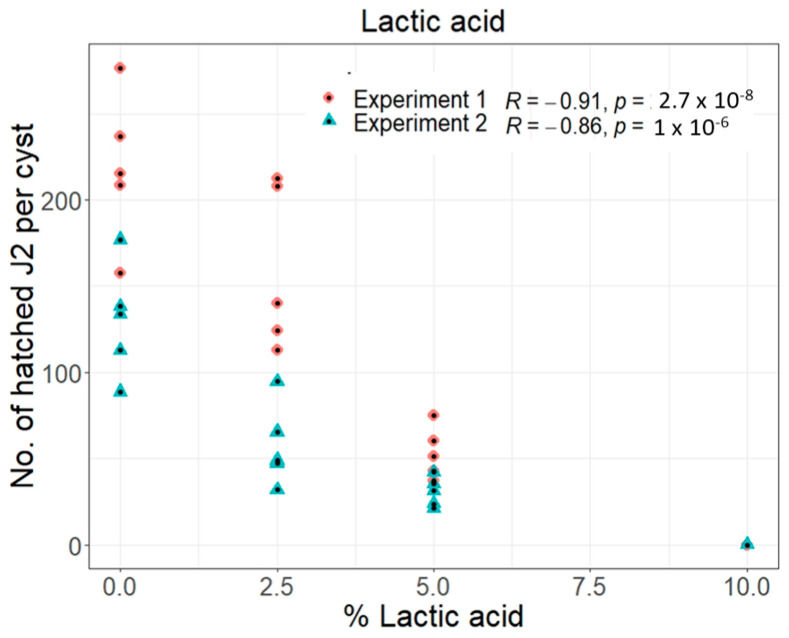
Hatching test for *Globodera pallida* exposed to L-lactic acid. Cysts were treated with 0%, 2.5%, 5% and 10% L-lactic acid for seven days, n = 5. Shown is the Pearson correlation coefficient for the total number of hatched second stage juveniles (J2s) per cyst and the L-lactic acid concentration of two independent experiments.

Bacteria in the soil can produce lactic acid during the inundation process under anaerobic conditions, leading to a decrease of the pH in the Brukner and sedimentation basin. To test whether L-lactic acid solutions with low pH-values have an impact on the hatching of PCN, 20 *G. pallida* cysts were incubated in autoclaved tap water (control, 0%), 2.5% (pH 2.23), 5% (pH 2.07) and 10% (pH 1.92) L-lactic acid for 7 days (5 replicates, Figure 3). The lowest activity was seen after seven days in 10% L-lactic acid solution (~0%), followed by 5% solution after seven days (24%) (Figure 3). A clear negative correlation in the hatching of second stage juveniles (J2s) was seen with increasing L-lactic acid concentrations after seven days.

Our results show that exposure of the cysts to high concentrations of L-lactic acid is necessary for the efficient inactivation of *G. pallida*.

#### 3.1.4. Slaked Lime Treatment Reduces the Hatching from *G. pallida* Cysts

Calcium hydroxide (slaked lime) is a chemical that is used in the sugar beet processing cycle to remove hemicellulose fractions from the sugar juice to facilitate subsequent crystallisation. To minimise sugar losses through enzymes that are released by microorganisms and can lead to unwanted breakdown of sucrose, slaked lime is added to the wash water to inhibit enzymatic activity at the beginning of the sugar beet processing.

**Figure 4:**
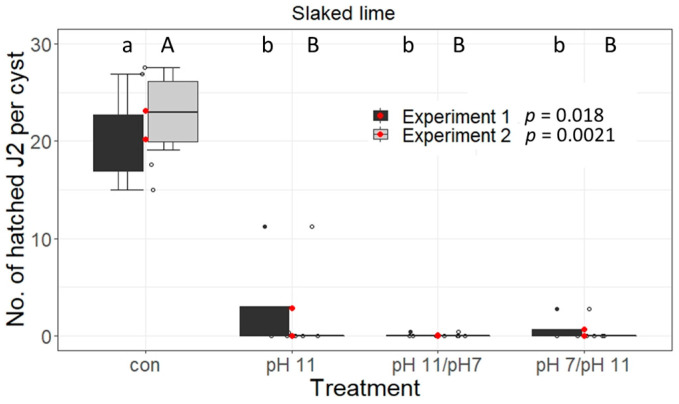
Hatching test for *Globodera pallida* exposed to slaked lime. Shown is the total number of hatched second stage juveniles (J2s) per cyst over a period of seven weeks from two independent experiments. Beforehand, cysts were embedded in 150 mL of soil and either treated with 200 mL of slaked lime for four days (pH 11), two days with 200 mL of slaked lime and two days with 200 mL of water (pH 11/ pH 7) or two days with 200 mL of water and two days with 200 mL of slaked lime (pH 7/ pH 11), nontreated reference cysts served as controls (con). Data were analysed using non-parametric Kruskal-Wallis tests with pairwise Wilcoxon post hoc tests. Box-Whisker-plots display median and mean (red dots) values of four replicates with 10 cysts each. Different letters indicate significant differences between the treatments of the respective experiment.

To simulate exposure of *G. pallida* PCNs to slaked lime, four gauze sachets containing 15 cysts each were exposed to either sandy soil or clay loam soil, saturated with slaked lime (pH 11) for four days. Some cysts were then additionally incubated in soil with pH 7 for another two days. The reverse setting (four days at pH 7, followed by two days at pH 11) was tested in parallel. We show that incubation of PCN in slaked lime only reduced the hatching activity (representing the viability of PCN eggs and juveniles) by 85–100%. Further reduction could be achieved by additional exposure to pH 7 (Exp.1 and Exp.2: 0 hatched J2s per cyst) but the difference was not statistically significant (Figure 4).

### 3.2. Treatment of PCN and BCN Cysts in Sedimentation Basins during Operational Sugar Beet Processing

#### 3.2.1. Inactivation of *G. pallida* and *H. schachtii* Cysts in a Sedimentation Basin System

To confirm our in vitro experiments, we introduced *G. pallida* into the sedimentation basin system of a sugar beet factory in Uelzen (Lower Saxony, Germany) under operating conditions, as described in the Methods (Section 2.2). We set up two sets of experiments in two consecutive years (2018/2019 and 2019/2020) to study the influence of the Brukner and/or sedimentation basins on PCN and BCN (Figure 1c).

**Figure 5:**
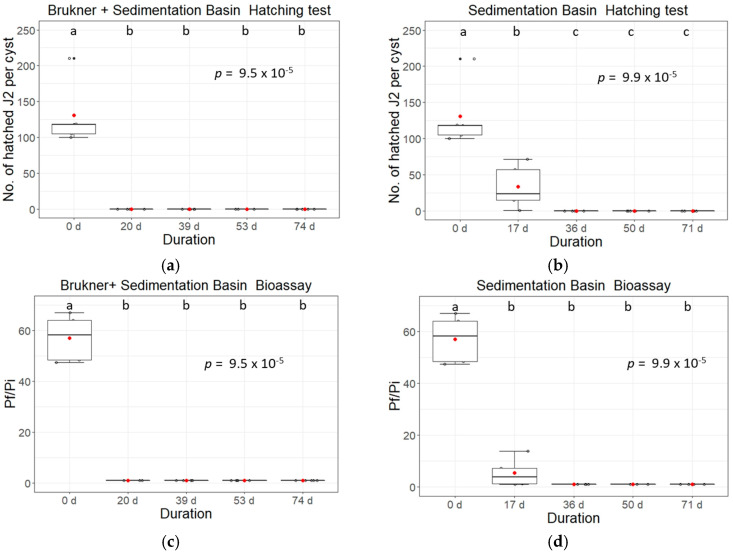
Hatching and reproduction rates of inundated and control cyst samples of *Globodera pallida* placed in sedimentation basin system during sugar beet processing campaign 2018/2019. (**a**,**b**) Total number of hatched second stage juveniles (J2s) per cyst. Cysts with previous treatment for three days in Brukner basin (**a**), or without pre-treatment (**b**) placed for 17, 36, 50 or 71 d in the sedimentation basin. Reproduction rates analysed in bioassays of *Globodera pallida* stored in inundation basins in 2018/2019 (**c**,**d**). Shown is the P_f_/P_i_ ratio of PCN added to the susceptible potato cultivar “Desiree”; cysts were counted 15 weeks after the potato planting. Pi cysts were treated before (**c**) for three days in a Brukner basin followed by 17, 36, 50 or 71 d in the sedimentation basin, or (**d**) placed for 17, 36, 50 or 71 d in the sedimentation basin. Data were analysed using non-parametric Kruskal-Wallis tests with pairwise Wilcoxon post hoc tests. Box-Whisker-plots display median and mean (red dots) values of five replicates with 20 cysts each. Different letters indicate significant differences between treatments.

**Figure 6:**
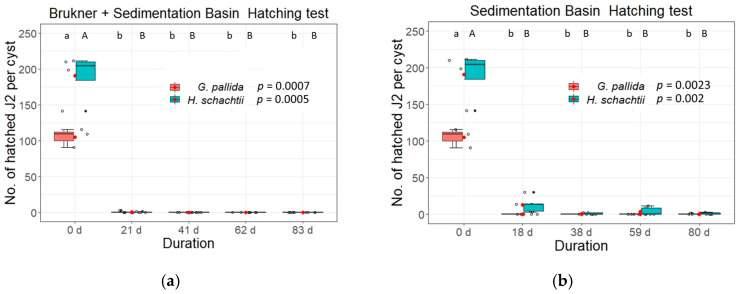

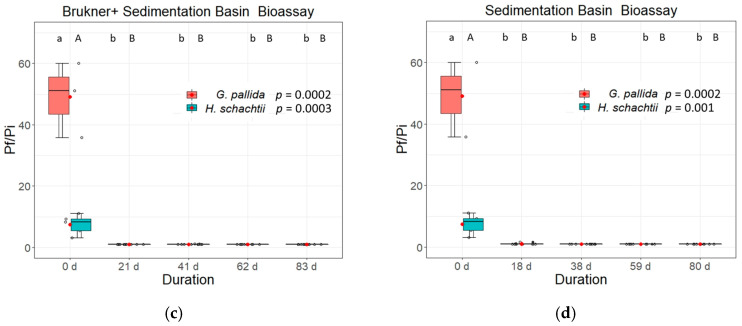
*Globodera pallida* and *Heterodera schachtii* hatching ratios of second stage juveniles (J2s) from cysts placed in inundation basins in campaign 2019/2020 (**a**,**b**). Shown is the total number of hatched second stage juveniles (J2s) per cyst pre-treated for three days in a Brukner basin (**a**) or placed immediately into the sedimentation basin (**b**). Samples (**a**,**b**) were placed into the sedimentation basin for 18, 38, 59 or 80 d. Reproduction rates of *Globodera pallida* and *Heterodera schachtii* placed in inundation basins in 2019/2020 (**c**,**d**). Shown is the P_f_/P_i_ ratio of cysts added to a susceptible host plant (PCN: potato cultivar “Desiree”; P_f_ cyst counting 15 weeks after planting; BCN: oilseed radish cultivar “Siletina”; P_f_ cyst counting 10 weeks after sowing). P_i_ cysts were treated (**c**) for three days in a Brukner basin followed by 18, 38, 59 or 80 d in the sedimentation basin, or (**d**) placed for 18, 38, 59 or 80 d in the sedimentation basin. Data were analysed using non-parametric Kruskal-Wallis tests with pairwise Wilcoxon post hoc tests. Box-Whisker-plots display median and mean (red dots) values of five replicates with 20 cysts each. Different letters indicate significant differences between treatments of the respective experiments.

During the first experiments (period of 2018/2019), we observed a sharp drop in hatching of second stage juveniles in *H. schachtii*, even in the control group (data not shown). We observed that sampled, soil-free BCN cysts dried out during transport from the sedimentation basin to the laboratory, leading to loss of viability. We tested the transport of gauze sachets in 1 g of moist loess-clay or wrapped the sachets in moist kitchen roll. The optimal conditions for survival of the eggs/juveniles in the cysts were achieved by wrapping the gauze sachets in paper tissue moistened with distilled water and shipping them overnight in a cooled, closed container. These optimised shipping conditions were then used for samples from *H. schachtii* in the second period (2019/2020) of our experiments. Results for *G. pallida* could be obtained for both times. Analysis of *G. pallida* cysts per time point and condition showed that incubation of PCN in the sedimentation basin reliably inactivated the hatching of second stage juveniles (J2s) and the reproduction of females after 5.5 weeks. In 2018/2019 we observed a 25% hatching rate and a P_f_/P_i_ ratio of 5.5 in samples that were exposed for 2.5 weeks in the sedimentation basin (Figure 5b,c). An additional three days of pre-exposure to the Brukner basin shortened the time of inactivation to three weeks (Figure 5 and Figure 6).

Similarly, inactivation of *H. schachtii* could be observed three weeks after a 3-day pre-incubation in the Brukner basin (Figure 6a). Exposure of the BCN cysts to the sedimentation basin only prolonged the time until hatching inactivation to at least nine weeks (hatched J2s per cyst day 18: 12.9, day 38: 1, day 59: 4.2, and day 80: 1.2) (Figure 6b). Production of females in the bioassay was successfully inhibited after 5.5 weeks with or without pre-exposure to the Brukner basin (Figure 5c,d and Figure 6c,d).

**Figure 7:**
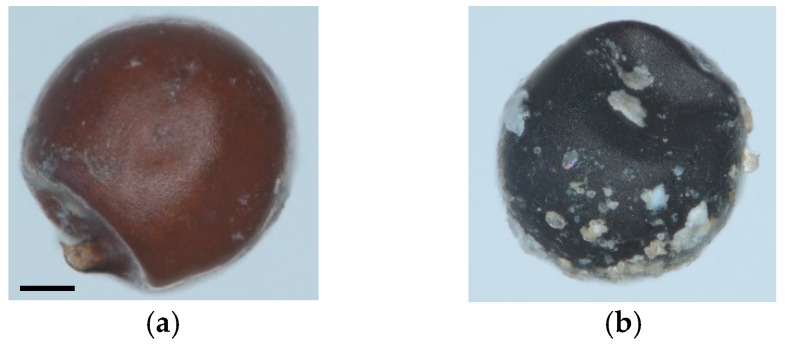
*Globodera pallida* cysts (**a**) before and (**b**) after a treatment for three weeks in the sedimentation basin. Pictures were taken with Axio Zoom V16, Carl Zeiss, Germany at 60× magnification. Scale bar shown under (**a**) measuring 100 µm.

To optically observe any changes in the cysts and egg/juvenile morphology, samples of *G. pallida* were analysed under the microscope at w3 after treatment in the sedimentation basin during the 2018/2019 time period. We noticed that the shape of the cysts was deformed, and the colour of the cyst wall changed from fawn brown (Figure 7a) to dark brown with white deposits (Figure 7b).

**Figure 8:**
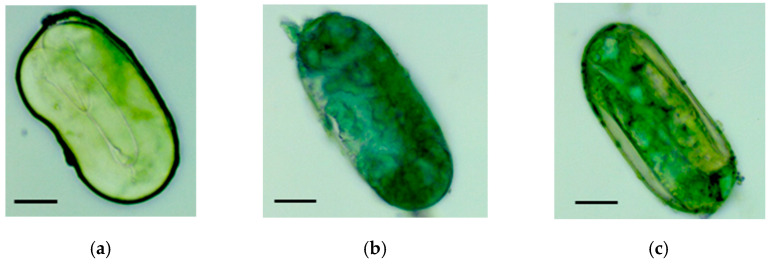
*Globodera pallida* eggs stained with Malachite green (**a**) before and (**b**,**c**) after three weeks of treatment in the inundation sedimentation basin. Pictures were taken with a Leica CTR 5500 microscope (Leica MZ APO, Leica Microsystems, Wetzlar, Germany) at 400× magnification. Scale bar shown under (**a**) measuring 20 µm.

Additionally, *G. pallida* eggs were damaged during the inundation process, illustrated by staining of eggs and the juveniles inside them with Malachite green, which can only penetrate into eggs without an intact eggshell (Figure 8b,c). Some of the eggs also showed a fungal structure (Supplementary Figure S4). In comparison, staining of the control cyst was much weaker and the juvenile structure with its prominent stylet could be observed (Figure 8a).

#### 3.2.2. Importance of the Brukner Basin on Inactivation of PCN and BCN Cysts

**Figure 9:**
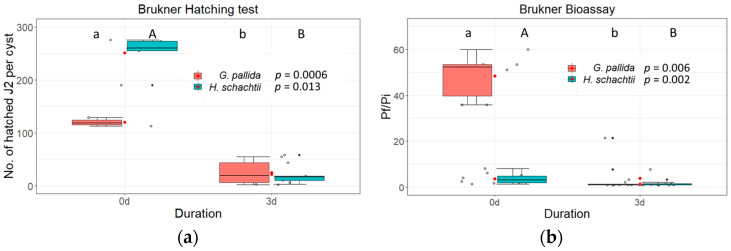
*Globodera pallida* and *Heterodera schachtii* cysts exposed in Brukner basin for three days in campaign 2019/2020. (**a**) Shown is the total number of hatched second stage juveniles (J2s) per cyst. (**b**) Reproduction rates of *Globodera pallida* or *Heterodera schachtii* stored in the Brukner basin for three days in 2019/2020. Shown is the P_f_/P_i_ ratio of cysts added to a susceptible host plant (PCN: potato cultivar “Desiree”; P_f_ cyst counting 15 weeks after planting; BCN: oilseed radish cultivar “Siletina”; P_f_ cyst counting 10 weeks after sowing). Data were analysed using non-parametric Kruskal-Wallis tests with pairwise Wilcoxon post hoc tests. Different letters indicate significant differences between the treatments of the respective experiments.

Since we saw a decreased viability of cysts when pre-incubation in the Brukner basin occurred, we wanted to test whether it was solely responsible for this effect. For this experiment, *G. pallida* and *H. schachtii* cysts were incubated in the Brukner basin for three days and then subjected to hatching assays and bioassays as described above. Both, i.e., hatching activity and production of females in the bioassay, were markedly decreased after this treatment. The number of hatched juveniles per cysts dropped down to 25 hatched J2s per cyst for *G. pallida* and to 21 for *H. schachtii*. The P_f_/P_i_ ratio lowered to one for *G. pallida* and for *H. schachtii* (Figure 9).

**Figure 10:**
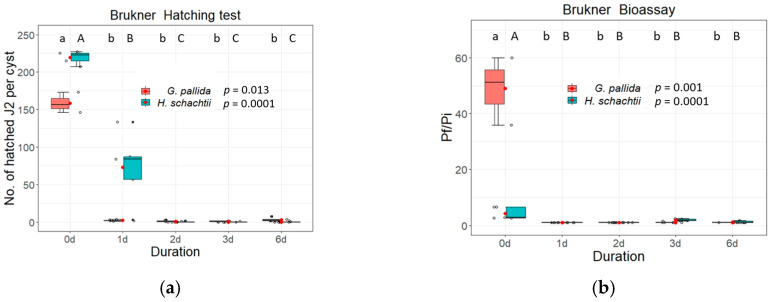
*Globodera pallida* and *Heterodera schachtii* cysts exposed in Brukner basin for 1, 2, 3 or 6 days in campaign 2019/2020. (**a**) Total number of hatched second stage juveniles (J2s) per cyst and (**b**) the P_f_/P_i_ ratio of PCN and BCN cysts added to a susceptible host plant (PCN: Potato cultivar “Desiree”; P_f_ cyst counting 15 weeks after planting; BCN: oilseed radish cultivar “Siletina”; P_f_ cyst counting 10 weeks after sowing). Data were analysed using non-parametric Kruskal-Wallis tests with pairwise Wilcoxon post hoc tests. Box-Whisker-plots display median and mean (red dots) values of four replicates with 20 cysts each. Different letters indicate significant differences between the treatments of the respective experiments.

To further specify the efficiency of inhibition in the Brukner basin we performed additional analyses on days 1, 2, 3 and 6 (Figure 10). *Heterodera schachtii* still showed some hatching activity after day 1 (73 hatched J2s per cyst), but the bioassay showed no female production on day 1 (P_f_/P_i_ ratio: 1). Slightly increased P_f_/P_i_ ratios were found at day 3 (P_f_/P_i_: 1.9) and at day 6 (P_f_/P_i_: 1.2). In contrast, *G. pallida* was already strongly inhibited after one day of incubation in the Brukner basin (2 hatched J2s per cyst).

## 4. Discussion

The inundation of tare soil in sedimentation basins may play a crucial role in the inactivation of nematode cysts and could be an easy and cost-effective way to disinfest re-usable tare soils. In our study, we analysed different steps of the processing cycle (pH changes, soil type, and exposure time) to determine the minimal requirements needed for inactivation of nematode cysts during inundation. As a model organism, we used the PCN *Globodera pallida* and confirmed our results by analysing the response of BCN *Heterodera schachtii* to inundation. Transport conditions from the test site to the laboratory are very important; we found that *H. schachtii* was more sensitive to dry conditions during transport than *G. pallida*. Therefore, this should be considered for different cyst nematodes species in experiments.

During the processing of tare soil in the sedimentation basins, cysts are exposed to different pH-values, which are alkaline in the Brukner basin and slightly acidic in the subsequent sedimentation basin. Therefore, we incubated *G. pallida* cysts for 24 h in KOH solutions with pH-values between 7.2 and 12 but did not see an impact on hatching activity. In the sugar factory fluctuations of the pH-values occur during the processing, first when slaked lime (pH 11) is added to alkalise the wastewater–tare soil mixture, and second when the mixture is stored in the sedimentation basins (pH 6). Slaked lime itself or the pH-changes could have an impact on the hatching activity of pest nematode cysts. We showed in a laboratory setting that incubation of *G. pallida* cysts for four days in slaked lime or when the cysts experienced pH-changes either from neutral to basic (normal process) or vice versa (recreating system cleaning processes, when the pipes are flushed with a pH 5.5 solution), hatching was severely impaired. These results were confirmed in our basin experiment under operating conditions in a sugar-processing factory. There, cysts were incubated for three days in the Brukner basin (pH~11), followed by prolonged inundation in the sedimentation basin (pH~6), which abolished the hatching activity and reproduction of *G. pallida* (Figure 5a,c and Figure 6a,c, Supplementary Figure S5c) and *H. schachtii* (Figure 5c,e) and destroyed the physiognomic structure of the eggs, as confirmed by visual inspection (Figure 8). In accordance with our results, Spaull and colleagues showed a 57% reduction of the viability of *G. pallida* cyst eggs in raw sewage after a 24 h treatment with 0.32 g of lime powder, which increased the pH in the sample from 5.5 to 11 [23]. Similar to our results, an extended incubation time decreased the hatching activity further.

We show that the Brukner basin apparently plays an important role in the inactivation of nematode cysts, but the usual time of tare soil resting in the Brukner basin during normal operation of the sugar beet factory is one to two days, which is shorter than our test conditions of three days. Therefore, we also tested the hatching activity and reproduction after incubation in sedimentation basins only (Figure 5b,d and Figure 6b,d, Supplementary Figure S5a,b,d,f). Without the Brukner treatment, the inactivation of pest nematode cysts under absence of oxygen at pH ~6 in the sedimentation basin took much longer in 2018/2019 with a rate of viable eggs and juveniles in some replicates higher than 60% (≥3 weeks, Figure 5b). Comparable results have been reported, where a four-week inundation in a flooding sedimentation basin resulted in a 90% reduction in the vitality of *G. pallida* nematode cysts, but viable eggs were still present after five months [8]. Another study showed a reduction of 90% and 99% egg viability after 120 days and six months incubation, respectively [10].

In parallel to hatching experiments, we also did bioassays to determine the inhibition of female development. Inundation of cysts with Brukner treatment efficiently inhibited the reproduction of cysts (Figure 5 and Figure 6, Supplementary Figure S5e). Our results highlight the importance of the Brukner basin for cyst inactivation during the inundation of tare soil, and our results further indicate that a prolonged exposure to the alkaline conditions in the Brukner basin could further enhance the disinfestation effects (see Figure 9 and Figure 10).

During inundation, aerobic/anaerobic conditions or additional toxic metabolites produced by microorganisms could have an impact on the viability of nematode cysts. We have shown that PCN cysts incubated in dry soil compared to wet soil showed higher viability (Figure 2), which could be due to access to oxygen. Dry cysts, partially filled with air, might float on the surface of flooding sedimentation basements, providing access to oxygen. During the inundation experiment in 2018/2019, we monitored the oxygen levels at the depth of cyst containers and found that oxygen was depleted throughout the entire incubation period in the sedimentation basin.

Anaerobic conditions and the different pH-values in the Brukner and sedimentation basins might favour specific microbial communities, which could then directly affect the nematode eggs or produce metabolites that are toxic to pest nematode cysts. Seo and colleagues reported the successful inactivation of *Meloidogyne incognita* juveniles on plants using mixtures of organic acids, among them lactic acid [24]. We showed that L-lactic acid solutions, which can denature enzymes and provides an acidic milieu, was indeed able to inactivate the resting PCN eggs under laboratory conditions. The L-lactic acid concentration in the basin was 0.6%, whereas, at the exit of the Brukner basin, 1.4% L-lactic was measured in a sedimentation basin in one of the companies’ factories in 2021 (Supplementary Table S1). We started with a 2.5% lactic acid concentration in the lab experiment and already saw a reduction in hatching activity. The size of the influence of the L-lactic acid under real conditions is difficult to estimate. We also only considered a seven-day period in the lab, while in the sedimentation basin, the incubation period is much longer. The results from the basin also showed that carbon hydrates, such as sucrose, glucose and fructose, had disappeared in the sedimentation basin, while Acetic acid, Butyric acid and Propionic acid accumulated (Supplementary Table S1) indicating an effect of microbial activity.

Furthermore, the microscopic analysis of eggs from the sedimentation basin (acidic conditions) showed associated fungal structures, indicating a direct interaction with the eggs. It is known from field cyst populations that antagonistic fungi can suppress nematode abundance, e.g., by producing lytic enzymes as shown for *Verticillium* sp. isolated from *H. glycines* and *H. avenae* cysts [25,26]. A recent study on *H. schachtii* also showed that *Exophiala* sp., *Pochonia chlamydosporia* and *Pyrenochaeta* sp. are frequently found as egg parasitising fungi [27].

The growth and development of microbial communities can be promoted by the presence of organic material that can lead to fermentation processes. The organic components mixed with soil tares during sugar beet processing are easily metabolised material, such as short-chained carbohydrates, whereas potato tare soils contain lower amounts of organic material. The additional specific introduction of organic material rich in carbon hydrates could be used to trigger the production of lactic acid, which could accumulate to support the inundation process. Ebrahimi et al. showed in 2 L bucket experiments that potato tare soil supplemented with steamed potato peel as a waste product in potato processing decreased the egg viability of *G. pallida* during inundation by 20% after two weeks and by 100% after four weeks, compared to potato tare soil only [28]. In contrast, Spaull et al. found a 99.7% inhibition of cyst eggs after a two-week incubation period in soil without organic supplements [5]. We also infer from our microflooding experiments (Figure 2) that the type of soil in the sedimentation basins is not important for the effectiveness of the inundation process.

The odour nuisance of fermentation processes needs to be considered. In addition, the installation costs of a sedimentation basin might appear high, but they are nonrecurring expenses. Where other disinfestation methods include regular high costs in terms of energy supply, inundation basins will pay off in the long term and allow larger quantities of tare soil to be processed each time. In addition, other soil-borne pathogens could be disinfected by inundation. In 2012, Molendijk and colleagues provided a detailed description on the effectiveness of inundation to kill off *Verticillium dahlia* in a microcosm experiment in the Netherlands by 84% [29].

In conclusion, the inundation process of tare soil throughout sugar beet processing efficiently inactivates PCNs and BCNs. In our study, we highlight the role of the presedimentation Brukner basin, which enables the exposure of the cysts to different conditions, such as varying pH-values or toxic metabolites produced by microorganisms. Therefore, inundation, as already performed in the sugar beet processing industry, would be a valuable measure to disinfest successful tare soils in the potato processing industry from *Globodera* spp. cysts.

## Figures and Tables

**Table 1:** Hatching activity of *Globodera pallida* exposed to water and KOH solutions with pH 7.2, 7.8, 9, 10, 11 or 12 for 72 h. Shown are the numbers of hatched second stage juveniles (J2s) per cyst, n = 3, one-way Anova *p* = 0.16. Different letters indicate significant differences between the treatments.

pH-Value	No. of Hatched j2 per Cyst
Autoclaved tap water (7.2)	103.3 ^a^
7.8	98.2 ^a^
9	103.4 ^a^
10	100.5 ^a^
11	98.6 ^a^
12	131.6 ^a^

## Data Availability

The data presented in this study are available on request from the corresponding author.

## Supplementary Materials

The following supporting information can be downloaded at: https://www.mdpi.com/article/10.3390/life13010057/s1, Figure S1: pH-values in the sedimentation basin, Figure S2: Oxygen content in the sedimentation basin, Figure S3: Temperature curve in the sedimentation basin, Figure S4: *Globodera pallida* eggs with fungal structures, Figure S5: *Globodera pallida* and *Heterodera schachtii* hatching test and bioassay for Basin 2 in 2018/2019 and 2019/2020, Table S1: Organic acids and carbon hydrates in the sedimentation basin.
