# Supplementary material for: Tare Soil Disinfestation from Cyst Nematodes Using Inundation
Source: Life (Basel). 2022 Dec 24;13(1):57. doi: 10.3390/life13010057 (PMC9862421; doi:10.3390/life13010057)
Supplement: Supplementary file 1 [file life-13-00057-s001.zip › life-2078008-supplementary.pdf]

Supplemental data

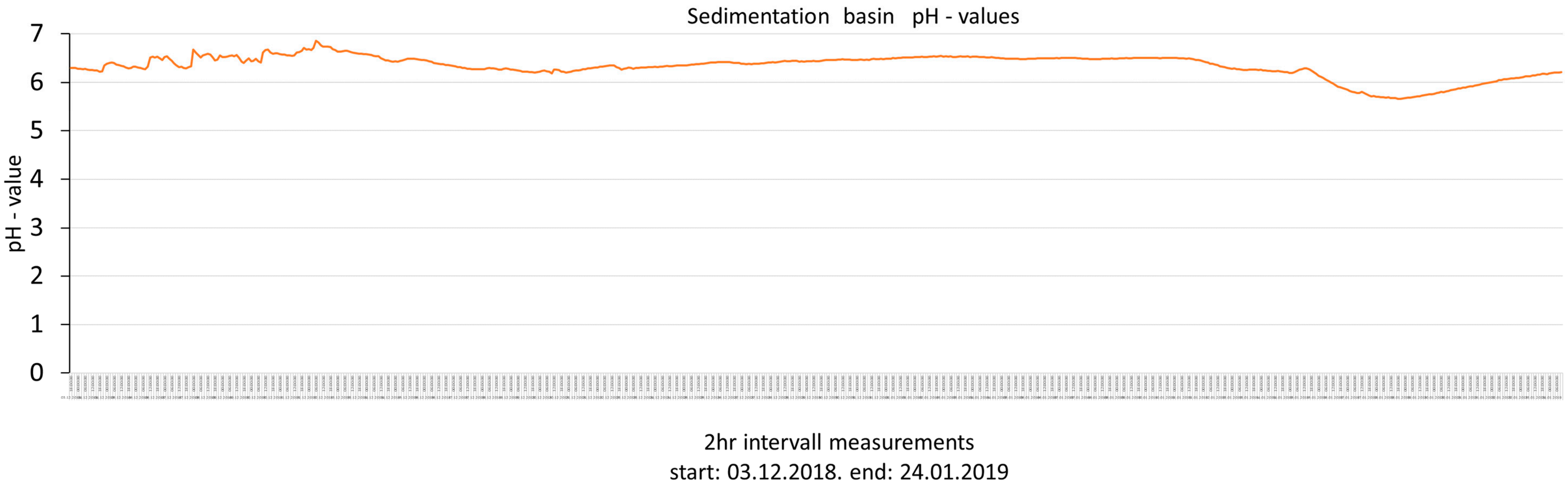

Supplementary Figure S1: pH-values in the sedimentation basin recorded in campaign 2018/2019.

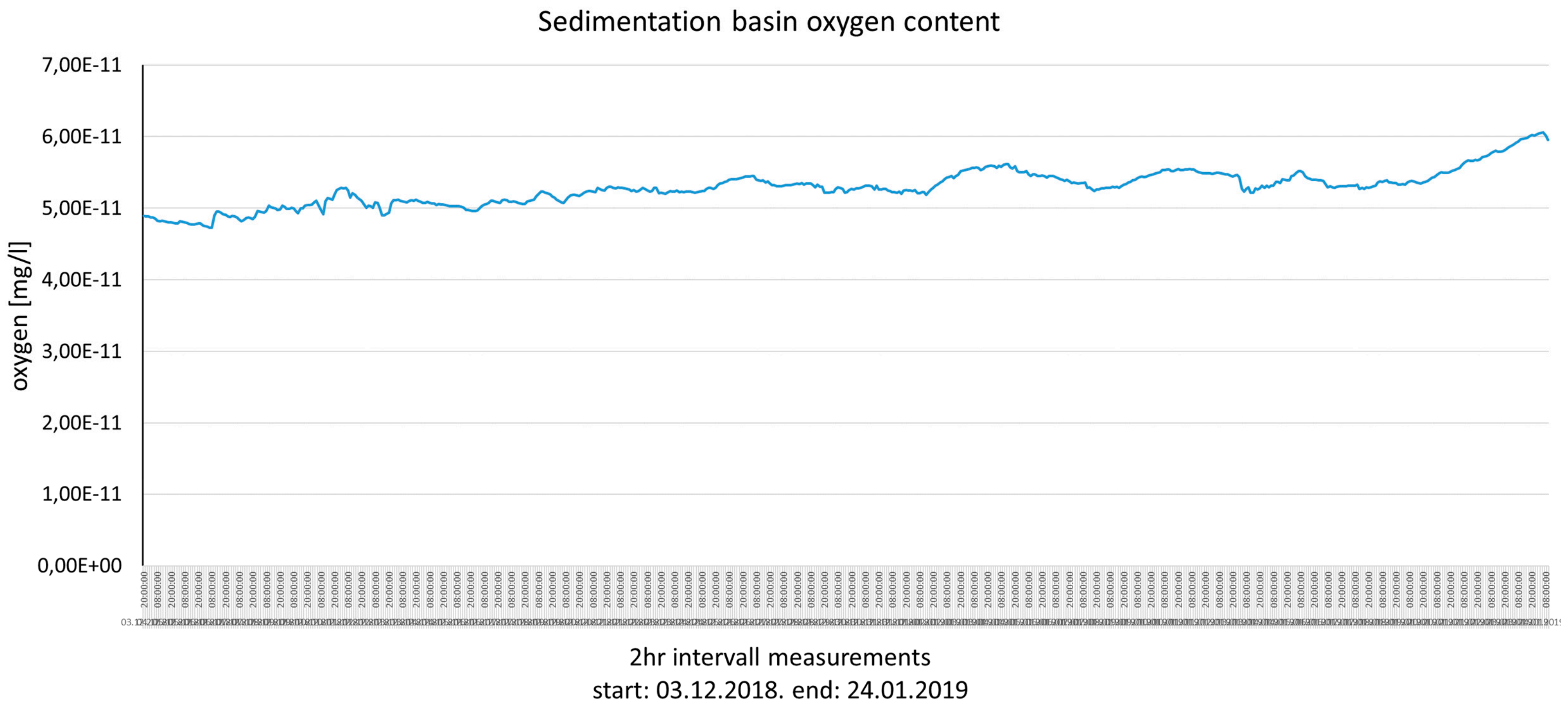

Supplementary Figure S2: Oxygen content in the sedimentation basin recorded in campaign 2018/2019.

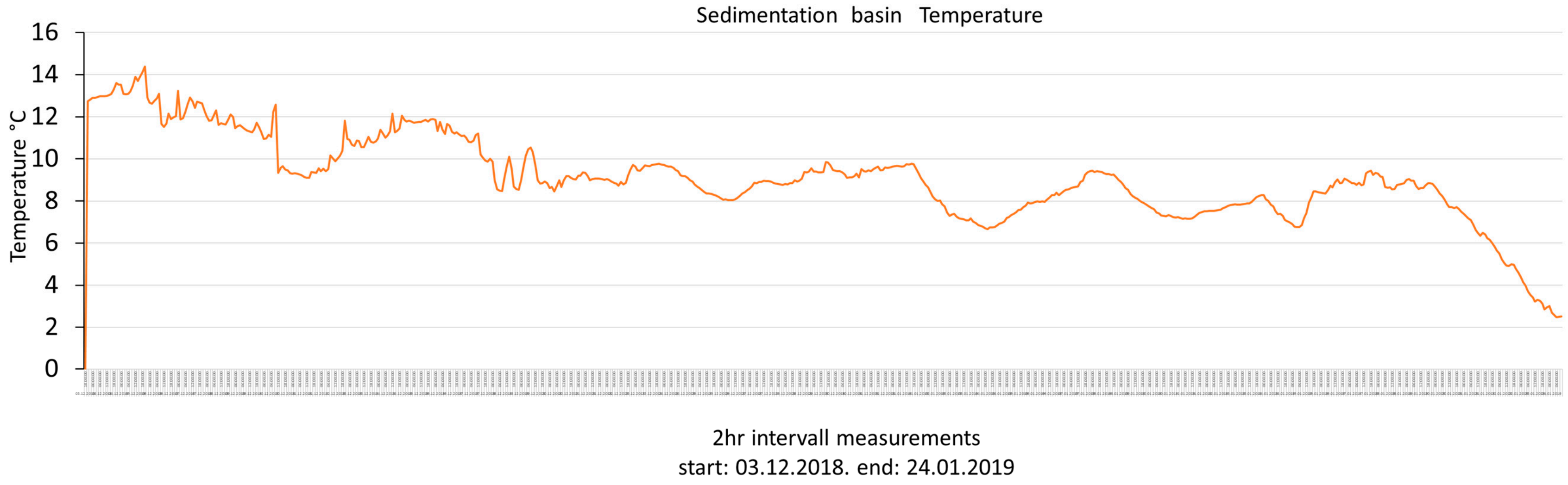

Supplementary Figure S3: Temperature curve in the sedimentation basin recorded in campaign 2018/2019

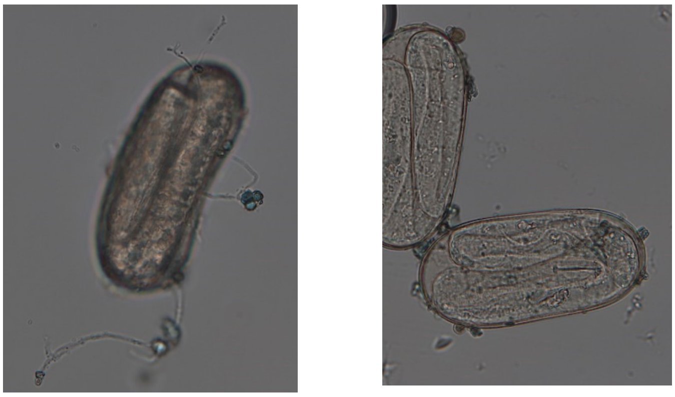

(a) **(b)**

Supplementary Figure S4: *Globodera pallida* eggs with fungal structures after a three weeks exposure in the inundation sedimentation basin (a, b). Pictures were taken with a Leica CTR 5500 microscope at 400x magnification

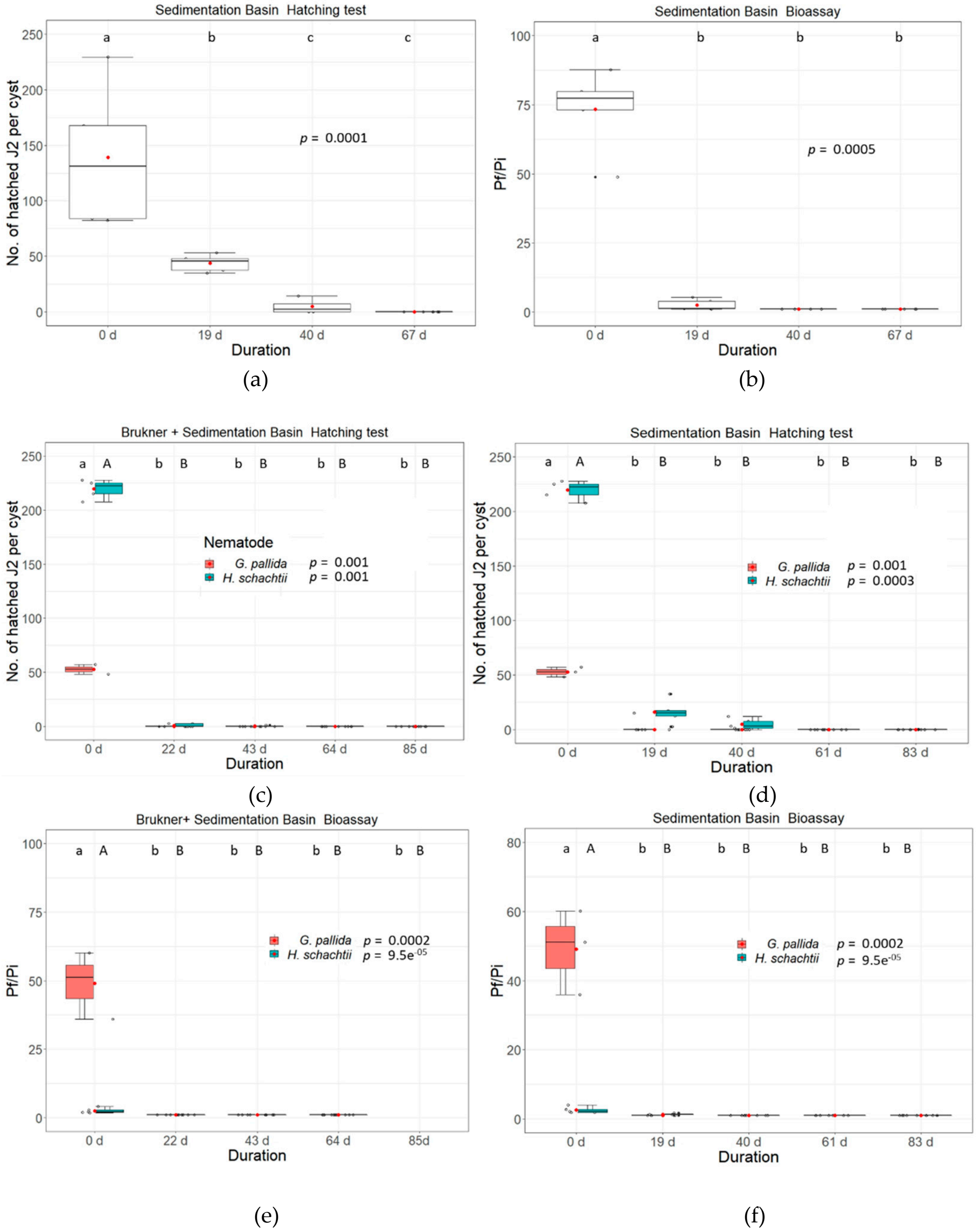

Supplementary Figure S5: Hatching ratios and bioassay for *Globodera pallida* from cysts placed in inundation basins in campaign 2018/2019 (a-b); *Globodera pallida* and *Heterodera schachtii* hatching ratios of juveniles (J2) (c-d) and bioassay (e-f) from cysts placed in inundation basins in campaign 2019/2020.

Supplementary Table S1: Organic acids and carbon hydrates content in the Sedimentation basin (six samples from different sites in the basin), at the exit of the Brukner basin and at the exit of the Sedimentation basin. Data were obtained in 2021 from a sugar factory similar to the one where the inundation experiments took place in 2018/2019 and 2019/2020.

			**Exit Brukner**	**Sedimentation basin**						**exit Sedimentation basin**
**Parameters**	**Method**	**Unit**	**Sample**	**Sample 1**	**Sample 2**	**Sample 3**	**Sample 4**	**Sample 5**	**Sample 6**	**Sample**
Sucrose	HPLC	mg L^{-1}	6529.9	<2	<2	<2	<2	<2	218.9	<2
Glucose	HPLC	mg L^{-1}	<2	<2	<2	<2	<2	<2	<2	<2
Fructose	HPLC	mg L^{-1}	<2	<2	<2	<2	<2	<2	<2	<2
Lactic acid	HPLC	mg L^{-1}	13721	53	2970	6217	9089	9490	9423	52
Formic acid	HPLC	mg L^{-1}	196	120	70	200	356	390	235	108
Acetic acid	HPLC	mg L^{-1}	994	2161	2597	2355	1983	1892	1988	2153
Propionic acid	HPLC	mg L^{-1}	438	3661	3193	2450	2028	2008	1735	3686
Butyric acid	HPLC	mg L^{-1}	642	6800	5570	4220	2804	2630	3051	6766
Isovaleric acid	HPLC	mg L^{-1}	8	37	27	17	9	9	12	36
Valeric acid	HPLC	mg L^{-1}	32	203	169	125	84	80	117	200
pH-value	Potentiometric		5.8	6.7	6.6	6.2	5.6	5.5	5.5	6.7
